# Quantitative Brain MRI in Congenital Adrenal Hyperplasia: *In Vivo* Assessment of the Cognitive and Structural Impact of Steroid Hormones

**DOI:** 10.1210/jc.2017-01481

**Published:** 2017-11-20

**Authors:** Emma A Webb, Lucy Elliott, Dominic Carlin, Martin Wilson, Kirsty Hall, Jennifer Netherton, Julie Reed, Tim G Barrett, Vijay Salwani, Jon D Clayden, Wiebke Arlt, Nils Krone, Andrew C Peet, Amanda G Wood

**Affiliations:** 1 Department of Endocrinology & Diabetes, Birmingham Children’s Hospital, Birmingham, United Kingdom; 2 Institute of Metabolism and Systems Research, University of Birmingham, Birmingham, United Kingdom; 3 Centre for Endocrinology, Diabetes and Metabolism, Birmingham Health Partners, Birmingham, United Kingdom; 4 University of East Anglia, Faculty of Medical and Health Sciences, Norwich, Norfolk, United Kingdom; 5 Institute of Cancer and Genomic Sciences, College of Medical and Dental Sciences, University of Birmingham, Birmingham, United Kingdom; 6 Centre for Human Brain Health and School of Psychology, University of Birmingham, Birmingham, United Kingdom; 7 School of Psychology, University of Birmingham, Birmingham, United Kingdom; 8 Department of Psychology, Birmingham Children’s Hospital, Birmingham, United Kingdom; 9 Department of Radiology, University Hospitals Birmingham NHS Foundation Trust, Birmingham, United Kingdom; 10 Developmental Imaging & Biophysics Section, UCL Great Ormond Street Institute of Child Health, London, United Kingdom; 11 Academic Unit of Child Health, Department of Oncology & Metabolism, University of Sheffield, Sheffield Children’s Hospital, Sheffield, United Kingdom; 12 School Life and Health Sciences & Aston Brain Centre, Aston University, Birmingham, United Kingdom; 13 Child Neuropsychology, Clinical Sciences, Murdoch Children’s Research Institute, Melbourne, Victoria, Australia

## Abstract

**Context:**

Brain white matter hyperintensities are seen on routine clinical imaging in 46% of adults with congenital adrenal hyperplasia (CAH). The extent and functional relevance of these abnormalities have not been studied with quantitative magnetic resonance imaging (MRI) analysis.

**Objective:**

To examine white matter microstructure, neural volumes, and central nervous system (CNS) metabolites in CAH due to 21-hydroxylase deficiency (21OHD) and to determine whether identified abnormalities are associated with cognition, glucocorticoid, and androgen exposure.

**Design, Setting, and Participants:**

A cross-sectional study at a tertiary hospital including 19 women (18 to 50 years) with 21OHD and 19 age-matched healthy women.

**Main Outcome Measure:**

Recruits underwent cognitive assessment and brain imaging, including diffusion weighted imaging of white matter, T1-weighted volumetry, and magnetic resonance spectroscopy for neural metabolites. We evaluated white matter microstructure by using tract-based spatial statistics. We compared cognitive scores, neural volumes, and metabolites between groups and relationships between glucocorticoid exposure, MRI, and neurologic outcomes.

**Results:**

Patients with 21OHD had widespread reductions in white matter structural integrity, reduced volumes of right hippocampus, bilateral thalami, cerebellum, and brainstem, and reduced mesial temporal lobe total choline content. Working memory, processing speed, and digit span and matrix reasoning scores were reduced in patients with 21OHD, despite similar education and intelligence to controls. Patients with 21OHD exposed to higher glucocorticoid doses had greater abnormalities in white matter microstructure and cognitive performance.

**Conclusion:**

We demonstrate that 21OHD and current glucocorticoid replacement regimens have a profound impact on brain morphology and function. If reversible, these CNS markers are a potential target for treatment.

## Teaser

Using quantitative neuroimaging and cognitive assessment, we demonstrate that 21OHD and current glucocorticoid replacement regimens have a profound impact on brain morphology and function.

Steroid hormones regulate central nervous system (CNS) development, exerting trophic effects on cell survival, differentiation, maturation, and synaptogenesis (1, 2). The most common variant of the inborn steroidogenic disorder congenital adrenal hyperplasia (CAH), 21-hydroxylase deficiency (21OHD), disrupts adrenal steroidogenesis at critical branch points of glucocorticoid (cortisol) and mineralocorticoid (aldosterone) synthesis. Reduced glucocorticoid feedback to the hypothalamo-pituitary axis results in increased central stimulation of adrenal steroidogenesis. This leads to increased adrenal androgen production and subsequent systemic androgen excess, glucocorticoid deficiency, and, in two-thirds of patients, clinically apparent mineralocorticoid deficiency (3). Medical treatment aims to replace the deficient hormones and to limit exposure to androgen excess. However, in clinical practice, patients are often exposed to both glucocorticoid and androgen excess (3). CAH therefore provides a pathophysiological model suited to study the impact of fluctuations in glucocorticoid and androgen exposure on human brain structure and function.

Qualitative evaluation of structural T2-weighted magnetic resonance imaging (MRI) studies in patients with CAH provides evidence of white matter hyperintensities (leukoaraiosis) in ≤46% of patients (4, 5), located predominantly in the temporal lobe, amygdala, hippocampus, periventricular matter, and corpus callosum. Leukoaraiosis is an uncommon incidental finding in healthy adults aged <45 years (6). The pathophysiology of leukoaraiosis remains poorly defined (7); however, the presence of local white matter abnormalities appears to indicate a global dysregulation of white matter structure, with changes in white matter microstructure in affected patients not limited to the areas of abnormality identified on visual inspection (8). Diffusion weighted imaging, a noninvasive MRI technique, provides quantitative indices of brain development, enabling the *in vivo* examination of white matter microstructure and characterization of white matter anatomy, including the degree of connectivity between different regions of the brain (9, 10). In pathologic conditions, diffusion anisotropy of water molecules is reduced because of altered diffusivity and disorganization of the white matter fibers. These measurements may become abnormal even before the lesion is morphologically apparent on conventional MRIs, helping both with early detection and with defining the extent of lesions. We hypothesized that diffusion tensor imaging (DTI) would identify significant reductions in quantitative indices of white matter microstructure in patients with CAH, including mean diffusivity, which reflects the degree of water mobility, and fractional anisotropy (FA), which is affected by axonal caliber, fiber density, and degree of myelination (11).

Measurements of *in vivo* human brain volumes can provide insights into the pathophysiology of patients’ cognitive abnormalities, with neuroimaging increasingly used in clinical trials as a biomarker to study the impact of treatment modifications. The extent of volumetric abnormalities present in patients with CAH has not previously been investigated with high-resolution MRI in conjunction with automated quantitative data analysis. In view of the high concentrations of androgen, mineralocorticoid, and glucocorticoid receptors in the hippocampus, amygdala, thalamus, brainstem, and cerebellum (2, 12–14), we hypothesized that patients with CAH would have localized volume loss in these regions.

Magnetic resonance spectroscopy (MRS), which enables the *in vivo* measurement of cerebral metabolites, provides a sensitive tool to measure the impact of changes in steroid hormone exposure on the brain. It has previously been used to investigate how excess glucocorticoid exposure affects cerebral metabolite concentrations in patients with Cushing disease (15). Patients with Cushing syndrome have reduced total choline, with normalization of the brain metabolite ratios after resolution of Cushing disease (15). MRS has not previously been performed in patients with CAH, in whom we hypothesized that total choline would be reduced.

Glucocorticoids exert an inverted *U*-shaped influence on human cognition, particularly on acquiring and consolidating memory (2), whereas androgens have an overall beneficial effect on cognitive control, verbal memory, and spatial cognition in humans (1, 13, 16). Although previous studies in patients with CAH have consistently identified impairments in short-term and working memory performance, thought to relate to excess glucocorticoid exposure, the relationship between these cognitive abnormalities and changes in brain structure has not previously been investigated (17). We hypothesized that neural abnormalities would correlate with cognitive performance and that increased glucocorticoid exposure would be associated with poorer performance on neuropsychometric tests and reductions in neural volumes, FA, and total choline.

## Subjects and Methods

### Patient selection

Adult women with CAH due to 21OHD diagnosed by hormonal and genetic testing were recruited from the endocrine clinic at University Hospital Birmingham and via the patient support group Children Living With Inherited Metabolic Diseases, Living With CAH. Local advertisements were placed at University Hospital Birmingham National Health Service Foundation Trust to recruit control subjects. Exclusion criteria for both groups were pregnancy, hypothyroidism, a medical condition known to affect cerebral anatomy, or metal in the body. Additional exclusion criteria for the control group were diagnosed psychiatric disorder, glucocorticoid use (ever), or diagnosed learning disabilities. The study was approved by the national research ethics committee of West Midlands, and all participants gave written informed consent before participation.

Ethnicity, age at diagnosis, handedness, highest educational level, employment category, and current glucocorticoid replacement dose were recorded. The genotype of patients with 21OHD was classified into genotype groups null, A, B, and C (18). In patients taking prednisolone instead of hydrocortisone, the equivalent dose was calculated by multiplying total prednisolone dose by four (19). Educational status was graded 1 to 6 based on the participant’s highest level of educational achievement [(1) *Completed primary school;* (2) *Completed middle school to age 16 years;* (3) *Completed high school to age 18 years;* (4) *Higher studies beyond school;* (5) *Degree;* and (6) *Postgraduate degree*]. All participants with CAH had fasting 9:00 to 11:00 am blood samples taken on the day of MRI acquisition before any medication for measurements of plasma renin concentration, serum 17-hydroxyprogesterone, dehydroepiandrosterone, androstenedione, and testosterone liquid chromatography and tandem mass spectrometry. Standing height was measured with a stadiometer.

### Psychometric assessment

Psychometric testing comprising Wechsler Adult Intelligence Scale (WAIS) IV and Wechsler Memory Scale (WMS) IV assessments was administered by a trained psychology research assistant (20, 21).

### Health-related quality of life and mood questionnaires

All study participants completed the World Health Organization Quality of Life and Hospital Anxiety and Depression Scale (HADS) questionnaires (22, 23). The World Health Organization Quality of Life questionnaire is an instrument to assess general well-being and comprises 26 items to broadly measure four domains: physical health, psychological health, social relationships, and environment. The HADS is a 14-scale questionnaire that is used to ascertain the levels of depression and anxiety a subject is experiencing.

### MRI acquisition

MRI was performed on a 3-T Philips Achieva scanner with a 32**-**channel head coil. One neuroradiologist (V.S.) blinded to the clinical data reviewed all images. DTI was performed with a monopolar Stejskal–Tanner sequence over 61 directions, with a single b = 0 image and b-factor of 1500 s/mm^2^. Data were collected in the axial plane, with an imaging matrix of 112 × 112 over 72 slices at an isotropic resolution of 2 mm. An echo time of 78 ms was used with a SENSE parallel imaging factor of 2. High-resolution three-dimensional T1-weighted images were acquired (repetition time = 8.40 ms, echo time = 3.8 ms, flip angle = 8°, field of view = 288 mm, 175 slices, 2 mm isotropic voxels). MRS data were acquired from an 18 × 18 × 18 mm voxel in the left mesial temporal lobe and a 20 × 20 × 20 mm voxel in the right parietal lobe. Point-resolved spectroscopy was performed at 3 T, with an echo time of 35 ms, a repetition time of 2000 ms, and 128 repetitions. A water unsuppressed MRS was acquired from the same voxel for water referencing.

### Image analysis

Diffusion-weighted images were initially processed with the Functional Magnetic Resonance Imaging of the Brain Software Library (http://www.fmrib.ox.ac.uk/fsl). Data were inspected for movement artifacts and then corrected for eddy current–induced distortions. Brain extraction and calculation of diffusion tensor FA and mean diffusivity maps were carried out with Functional Magnetic Resonance Imaging of the Brain Software Library tools. FA and mean diffusivity images were processed with tract-based spatial statistics and automated, observer-independent, voxel-by-voxel whole-brain between-group analysis (corrected for age) (24). Initially, every FA image was aligned to the FMRIB58 standard space FA map. Second, the mean FA image across subjects was created. The mean image was thinned and thresholded at a FA value of 0.2 to create a white matter tract skeleton representing the center of the tracts common to all subjects. FA data projected onto these skeletons was used in voxel-wise statistical comparisons with the threshold-free cluster enhancement option (corrected for multiple comparisons across space).

Amygdala, hippocampus, thalamus, cerebellum, brainstem, cerebrospinal fluid (CSF), and total brain volume were determined from the T1-weighted MRI with FreeSurfer, an automated segmentation tool (25). We selected the structures in the brain with the highest concentration of androgen, mineralocorticoid, and glucocorticoid receptors (2, 12–14). No other neural volumes were extracted from the FreeSurfer analysis to ensure that all analyses performed were hypothesis driven.

Spectroscopy data were analyzed using the TARQUIN algorithm version 4.3.7 to obtain concentrations for glutamate, total *N*-acetylaspartate and *N*-acetylaspartylglutamate (NAA), total choline (glycerophosphocholine and phosphocholine), total creatine (creatinine and phosphocreatine), and glutamine plus glutamate (Glx) (26). Because voxels tended to contain gray matter, white matter, and CSF in varying proportions, each voxel was segmented and the metabolite concentrations corrected for the differing water content (27).

### Statistical analysis

The current study was powered to detect a 20% difference [standard deviation (SD) 0.36] in amygdala volume (power = 0.85, *α* = 0.05) between patients and controls (28). Because the previous published neuroimaging study included only women with CAH (28), and there is a well-described sexually dimorphic pattern of brain development (29), all study recruits to the current study were female.

Baseline characteristics of the two groups were compared with unpaired Student *t* tests. Behavioral, quality of life (QOL), and cognitive assessment scores were compared between patients with CAH and controls with analysis of covariance corrected for education level. Correlations were performed to assess the relationships of markers of androgen and glucocorticoid exposure with psychometric assessment scores in patients with CAH. Data were analyzed in SPSS version 22.

Total brain, CSF volume, and cerebral metabolite concentrations were compared between patients with CAH and controls with analysis of covariance, with age as a covariate. For all other neural volumes, total brain volume was an additional covariate, and *P* values were adjusted to control for the false discovery rate (FDR) (30).

Partial correlations were used to assess the relationship between neural volumes and metabolite concentrations, when initial results indicated that there was a significant difference in neural volume and metabolite concentration between the two groups, and neuropsychometric scores in patients with CAH, with *P* values adjusted for FDR (30). For neural volumes, correlations were also controlled for total brain volume. Correlations were used to assess the relationship between markers of androgen and glucocorticoid exposure, neural volumes, FA, and mean diffusivity and metabolite concentrations (where there was a significant difference in neural volume and metabolite concentration between the two groups) only in patients with CAH, adjusted for FDR (30). Variables examined were those that showed a significant difference between patients with CAH and controls in group analyses.

## Results

### Study cohort characteristics

Nineteen adult women (mean 30.6 years, range 18 to 49 years) with 21OHD receiving glucocorticoids (median hydrocortisone dose 11.1 mg/m^2^ per day, range 10 to 13.8 mg/m^2^) and 19 healthy women (mean 32.8 years, range 21 to 50 years) were recruited between July 2015 and September 2016 (Table 1). All participants were right-handed, had no abnormal neurologic findings, and had completed mainstream schooling. All patients had 21OHD confirmed by molecular genetic analysis. Genotype groups null, A, B, and C contained 74%, 10.5%, 10.5%, and 5% of the patients, respectively (18), which means that most patients had a classic salt-wasting phenotype. One patient had nonclassic CAH. Median age at diagnosis with 21OHD was 2 weeks (range birth to 17 years). Sixteen of the 19 patients with CAH also had mineralocorticoid deficiency and were on fludrocortisone replacement (median dose 150 μg) at the time of assessment. Eight patients were taking prednisolone, and 11 were taking hydrocortisone; the largest dose was taken in the morning in all patients. Fifteen of 19 patients with CAH had been on the same glucocorticoid dose for ≥3 years. No patients with CAH were taking the oral contraceptive pill.

**Table 1.** Age, Auxology, Educational Level, Glucocorticoid Dose, and Fasting Steroid Hormone Concentrations

	Patients With CAH	Controls	*P*
Number	19	19	
Mean age, y (SD, SE)	30.6 (8.9, 2)	32.8 (8.5, 2)	0.4
Mean height, cm (SD, SE)	158.3 (8.3, 1.9)	164.9 (5.9, 1.4)	0.008*^a^*
Mean weight, kg (SD, SE)	77.1 (16.1, 3.7)	73.9 (17.8, 4.2)	0.57
Mean body mass index, kg/m^2^ (SD, SE)	30.9 (6.5, 1.5)	27.1 (6.1, 1.4)	0.08
Mean head circumference, cm (SD, SE)	55.2 (2.6, 0.6)	55.7 (2.1, 0.5)	0.6
Type 1 Chiari anomaly	4	0	
Mean educational level (graded 1 to 6) (SD, SE)	4.3 (1.3, 0.3)	4.3 (1.2, 0.3)	0.9

Patients With CAH Only	Median (Q1–Q3)	Reference Range	
Mineralocorticoid dose, μg/d	150 (100–237)	N/A	
Glucocorticoid equivalent dose, mg/m^2^/d*^b^*	11.1 (10–13.8)	N/A	
Glucocorticoid equivalent dose, mg/d	20 (16–25)	N/A	
Plasma renin concentration	27 (13–48)	1.8–59.4 ng/L	
Serum 17-hydroxyprogesterone	62 (2–168)	0.6–6 nmol/L	
Serum dehydroepiandrosterone	0.54 (0.2–2.6)	2.68–9.23 nmol/L	
Serum androstenedione	7.7 (3.2–15.3)	0.9–7.5 nmol/L	
Serum testosterone	1.9 (0.8–2.9)	<1.9 nmol/L	

Abbreviations: N/A, not applicable; SE, standard error.

^a^ *P* values remain significant after we controlled for FDR (30).

^b^ Hydrocortisone dose; in patients taking prednisolone instead of hydrocortisone, we calculated the equivalent dose by multiplying total prednisolone dose by 4 (19).

One patient with CAH was unable to complete the WAIS because of time constraints. One patient with CAH and one control did not tolerate the MRI scan but completed all other study components. MRI data quality was adequate in all subjects (V.S.). Temporal voxel MRS spectra were deemed to be suitable for analysis in 17 patients with CAH and 16 controls, and parietal voxel spectra were deemed to be suitable for analysis in 18 patients with CAH and 17 controls, as judged by visual inspection by two independent observers (A.C.P., D.C.).

### Psychometric assessment

The physical component of QOL was significantly lower in patients with CAH than in controls (*P* < 0.001) (Table 2). Patients with CAH scored higher on the depression component of the HADS questionnaire; however, no one scored in the clinical range (*P* = 0.02) (Table 2).

**Table 2.** HADS and World Health Organization QOL Scores in Patients With CAH and Controls Matched for Age, Sex, and Educational Status

	Patients With CAH	Controls	*P*
World Health Organization QOL questionnaire			
Number of participants	19	19	
QOL Physical	92 (80–108)	128 (116–132)	0.000001*^a^*
QOL Psychological	72 (60–96)	92 (88–100)	0.049
QOL Social Relationships	44 (36–52)	48 (40–52)	0.56
QOL Environment	116 (108–136)	124 (108–136)	0.37
QOL Total	90 (77–101)	108 (96–114)	0.001*^a^*
HADS questionnaire			
HADS Anxiety	7 (5–11)	6 (4–8)	0.4
HADS Depression	5 (3–7)	2 (1–3)	0.02*^a^*

Data presented as median (Q1 to Q3).

^a^ *P* values remain significant after we controlled for the FDR (30).

There was no significant difference in full-scale IQ between patients with CAH and controls. When compared with controls, patients with CAH had significantly lower working memory index (*P* = 0.04), processing speed (*P* = 0.03), digit span (*P* = 0.04), and matrix reasoning scores (*P* = 0.03) (Table 3).

**Table 3.** Cognitive Functional Assessment Scores in Patients With CAH and Controls Matched for Age, Sex, and Educational Status

	Patients With CAH	Controls	*P*
WAIS–Fourth Edition*^a^*			
Number of participants	18	19	
Full Scale IQ	94.5 (87–110)	110 (97–117)	0.09
Perceptual Reasoning Index	103.2 (92.5–119)	104 (98–115)	0.6
Verbal Comprehension Index	98.5 (82.5–110)	110 (87–112)	0.3
Working Memory Index	92 (76.2–105)	100 (100–114)	0.04*^b^*
Processing Speed Index	98.5 (89–113)	111 (102–120)	0.03*^b^*
Digit Span	7 (6–11)	10 (8–11)	0.04*^b^*
Arithmetic	9.5 (5.7–12.2)	11 (9–13)	0.1
Symbol Search	10.5 (8–12)	11 (10–13)	0.1
Coding	10 (7.8–12)	11 (10–15)	0.07
WMS*^a^*			
Number of participants	19	19	
Design 1 Scaled	9 (7–11)	11 (9–14)	0.02*^b^*
Design 2 Scaled	11 (9–12)	11 (10–16)	0.06
Visual Repetition Scaled 1	11 (8–13)	10 (8–12)	0.61
Visual Repetition Scaled 1	12 (9–15)	12 (10–14)	0.97
Content Scaled 1	10 (9–12)	12 (7–13)	0.4
Content Scaled 2	10 (8–12)	12 (10–15)	0.07
Spatial Scaled 1	10 (8–12)	12 (10–13)	0.13
Spatial Scaled 2	10 (9–13)	12 (9–15)	0.24
Auditory Memory Index	100 (93–113)	109 (94–118)	0.27
Visual Memory Index	105 (95–112)	110 (100–117)	0.22
Immediate Memory Index	102 (87–111)	109 (96–115)	0.16
Delayed Memory Index	104 (98–114)	115 (104–123)	0.14

^a^ Results presented as median (Q1 to Q3) corrected for educational level.

^b^ Although we had *a priori* hypotheses that working memory, processing speed, and digit span would be reduced in patients with CAH based on previous studies, these results did not remain significant after correction for false discovery rate (17, 32, 34).

### CNS abnormalities in patients with CAH

Four of 19 patients with CAH (21%) had a type 1 Chiari anomaly; brain MRI was otherwise normal on visual inspection in all subjects. In none of the study participants was a pituitary gland abnormality reported; however, specific pituitary views were not acquired.

**Figure 1.**
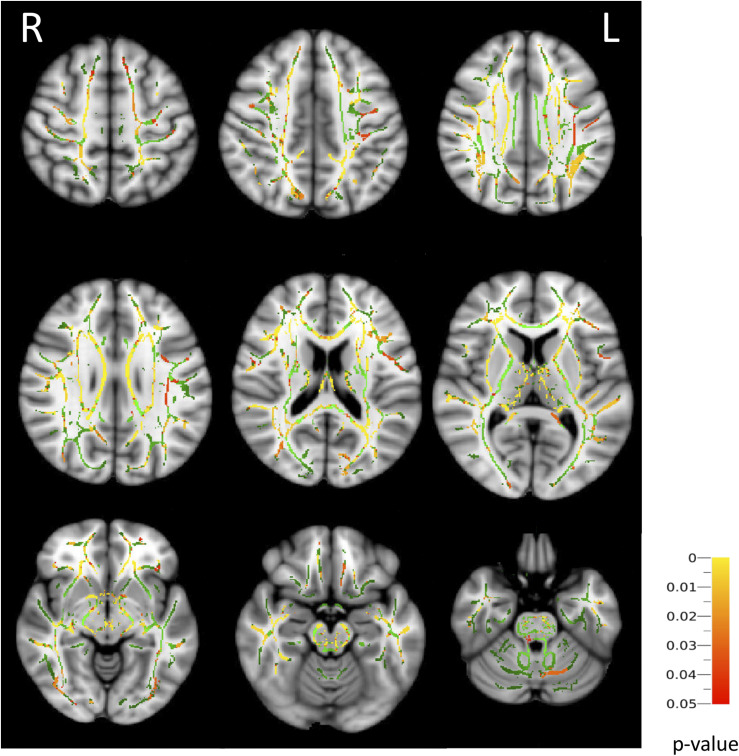
Association between CAH and FA (tract-based spatial statistics analysis comparing patients with CAH and healthy controls). Mean FA skeleton overlaid on the mean FA map. Regions of the mean FA skeleton in green represent areas where there were no significant differences in FA values in the patients with CAH and healthy controls. Areas in red and yellow are regions where the FA was significantly lower in the CAH group and can be observed bilaterally in the superior longitudinal fasciculus, inferior fronto-occipital fasciculus, corticospinal tract, uncinate fasciculus, cingulate gyrus, hippocampus, and corpus callosum (*P* < 0.05). FA is a dimensionless index.

Widespread reductions in FA were present in the superior longitudinal fasciculus, inferior fronto-occipital fasciculus, corticospinal tract, uncinated fasciculus, cingulate gyrus, hippocampus, and corpus callosum in patients with CAH (*P* < 0.05) (Fig. 1). Mean diffusivity was increased bilaterally in the superior and inferior longitudinal fasciculus, inferior fronto-occipital fasciculus, anterior thalamic radiation, corticospinal tract, uncinate fasciculus, cingulate gyrus, and hippocampus in patients with CAH (*P* < 0.05).

Although CSF volume was significantly higher in patients with CAH (*P* = 0.003), total brain volume was not significantly different between patients with CAH and controls. After correction for multiple comparisons, localized reductions in neural volumes were present in patients with CAH in the right hippocampus, left and right thalami, cerebellum, and brainstem (*P* = 0.028, 0.007, 0.008, 0.014, 0.03, respectively) (Table 4).

**Table 4.** Neural Volumes Determined From T1-Weighted MRI and FreeSurfer

	Patients With CAH	Controls	*P*
Number of participants	18	18	
Total brain volume (interquartile range)	1044732 (115246)	1069078 (86972)	0.47
CSF	1013 (914–1302)	839 (750–918)	0.003*^a^*
Left hippocampus	3792 (3611–4054)	4014 (3644–4271)	0.16
Right hippocampus	3744 (3639–3969)	3981 (3682–4272)	0.028*^a^*
Left amygdala	1509 (1406–1685)	1658 (1492–1848)	0.055
Right amygdala	1680 (1524–1785)	1718 (1488–1958)	0.6
Left thalamus	6318 (5894–6874)	6906 (6262–7578)	0.007*^a^*
Right thalamus	6240 (5689–6730)	6622 (6333–7079)	0.008*^a^*
Cerebellum (SD, SE)	113878 (106656–125254)	127499 (11484–134100)	0.014*^a^*
Brainstem	19145 (17569–20910)	20527 (19863–21794)	0.03*^a^*
Mesial temporal voxel metabolites			
Number of participants	17	16	
Glutamate	7.9 (6.2–10.3)	8 (4.9–10.8)	0.6
Total NAA	5.6 (4.6–6.3)	6.1 (4.9–7.2)	0.1
Total choline	1.9 (1.5–2.1)	2.3 (2–2.5)	0.001*^a^*
Total creatine	6.8 (5.8–7.9)	8 (7.6–8.9)	0.04
Glx	14.6 (11.2–18.3)	15 (11–16.9)	0.7
Parietal voxel metabolites			
Number of participants	18	17	
Glutamate	3.7 (2.9–4.4)	3.6 (2.4–4.5)	0.8
Total NAA	7.7 (7.2–8.8)	7.6 (7.3–8.6)	0.6
Total choline	2 (1.6–2.2)	1.9 (1.7–2)	0.5
Total creatine	6.4 (5.8–6.7)	6.1 (5.8–6.6)	0.6
Glx	4.5 (3.1–7.2)	6.1 (3–8.4)	0.7

Data presented as median (Q1 to Q3). Results corrected for age at scan and total brain volume (25) and spectroscopy-acquired CNS metabolite concentrations; data analyzed with TARQUIN (26).

Abbreviation: SE, standard error.

^a^ *P* values remains significant after we controlled for the FDR (30).

There were no significant differences in metabolite concentrations between patients with CAH and controls in the parietal voxel. In the mesial temporal voxel, total choline and total creatine were significantly lower in patients with CAH than controls (*P* = 0.001 and *P* = 0.04, respectively) (Table 4). Only total choline remained significantly lower after correction for multiple comparisons.

### Correlations between MRI findings and cognitive assessment scores in patients with CAH

**Figure 2.**
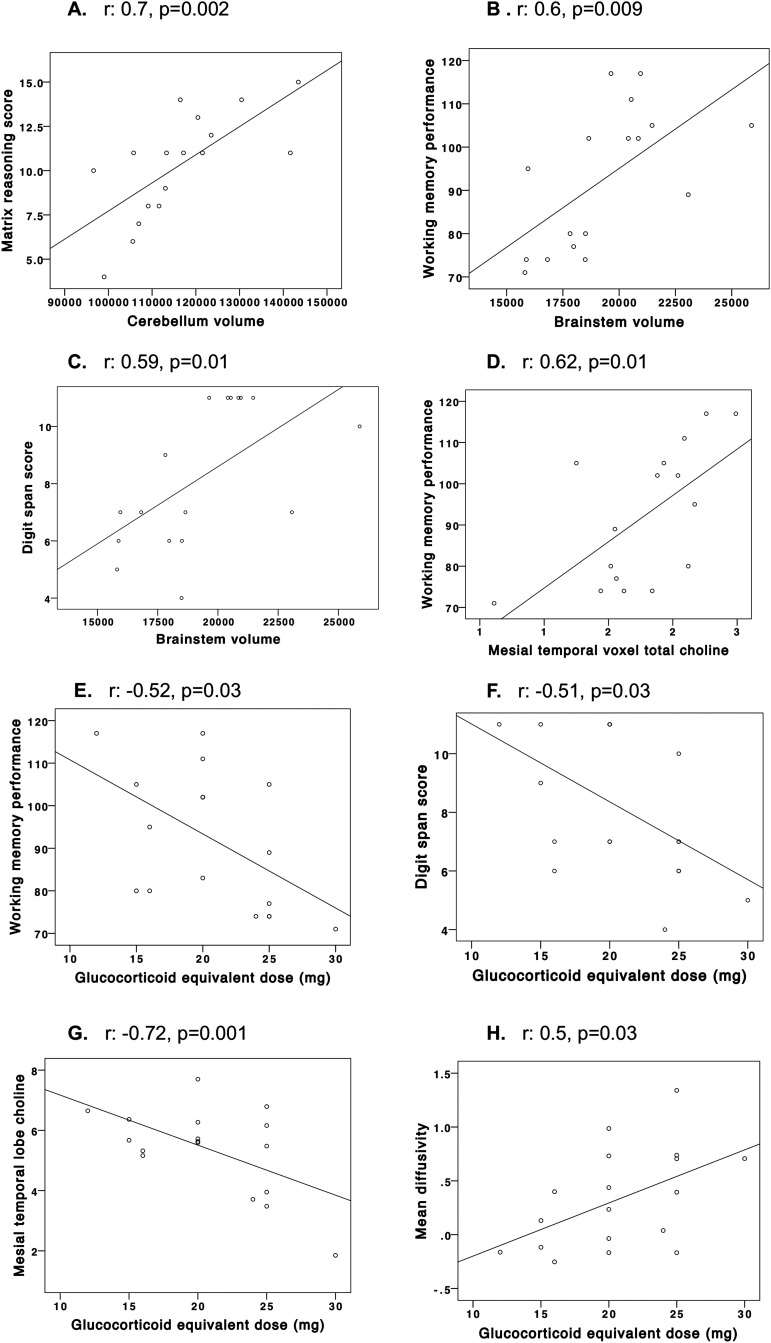
Associations between cognitive function scores and neural volumes, hydrocortisone equivalent dose, cognitive function scores, mesial temporal lobe total choline, and mean diffusivity in adults with CAH. Partial correlations were used to assess the relationship between neural volumes and metabolite concentrations and neuropsychometric test results for WAIS and WMS assessments in patients with CAH. For neural volumes, correlations were also controlled for total brain volume. Correlations were used to assess the relationship between markers of androgen and glucocorticoid exposure, neural volumes, fractional anisotropy, and mean diffusivity and metabolite concentrations, in patients with CAH. All *P* values were adjusted to control for the FDR (30). There were significant correlations between (A) cerebellar volume and matrix reasoning scores (*r* = 0.7, *P* = 0.002) and (B and C) brainstem volume, working memory performance, and digit span scores (*r* = 0.6, *P* = 0.009, *r* = 0.59, *P* = 0.01, respectively). (D) Spectroscopy-acquired left hippocampus total choline correlated significantly with working memory index in patients with CAH (*r* = 0.62, *P* = 0.01). (E and F) Increased glucocorticoid equivalent dose (mg) correlated significantly with reduced working memory (*r* = −0.52, *P* = 0.03) and digit span (*r* = −0.51, *P* = 0.03) scores. Higher glucocorticoid equivalent dose (mg) correlated significantly with (G) reduced mesial temporal lobe total choline (*r* = −0.72, *P* = 0.001) and (H) mean diffusivity (*r* = −0.5, *P* = 0.03).

There were significant correlations between cerebellar volume and matrix reasoning scores (*r* = 0.7, *P* = 0.002; Fig. 2A) and between brainstem volume, working memory performance, and digit span scores (*r* = 0.6, *P* = 0.009, *r* = 0.59, *P* = 0.01, respectively; Fig. 2B and 2C). Spectroscopy-acquired left mesial temporal lobe total choline correlated significantly with working memory index (*r* = 0.62, *P* = 0.01; Fig. 2D). There were no significant relationships between FA and mean diffusivity and cognitive assessment scores.

### Correlations between markers of glucocorticoid and androgen exposure, MRI findings, and cognitive assessment scores in patients with CAH

Increased glucocorticoid equivalent dose (mg) correlated significantly with reduced working memory (*r* = −0.52, *P* = 0.03) and digit span scores (*r* = −0.51, *P* = 0.03) (Fig. 2E and 2F). There were no significant relationships between markers of androgen exposure and psychometric assessment scores.

Higher glucocorticoid-equivalent dose (mg) correlated significantly with reduced mesial temporal lobe total choline (*r* = −0.72, *P* = 0.001; Fig. 2G) and mean diffusivity (*r* = −0.5, *P* = 0.03; Fig. 2H), suggesting that patients exposed chronically to higher glucocorticoid doses have greater reductions in mesial temporal lobe total choline and white matter microstructure, reflected by total choline concentration and mean diffusivity. There were no significant differences in any of the cognitive or MRI abnormalities identified between those taking prednisolone and those prescribed hydrocortisone. There were no significant associations between markers of androgen exposure, MRI, and cognitive findings.

## Discussion

Using multiple quantitative imaging modalities in conjunction with neuropsychological assessment enabled us to identify functionally significant biomarkers of the disease process (CAH) and treatment effects (steroid exposure) in patients with CAH. Patients had global abnormalities of cerebral white matter, with localized reductions in neural volumes in regions of the brain that have previously been documented to contain high concentrations of androgen, mineralocorticoid, and glucocorticoid receptors (2, 12–14). The mesial temporal lobe was affected bilaterally, with significant reductions in white matter microstructure, right hippocampal volume, and left mesial temporal lobe choline. Interestingly, although markers of androgen exposure did not relate to the identified CNS abnormalities, exposure to higher glucocorticoid doses was associated with significantly worse cognitive performance and abnormal mesial temporal lobe total choline and white matter mean diffusivity.

Concordant with finding of previous studies (17, 31), we found reduced working memory and digit span scores and impaired quality of life. Although we had *a priori* hypotheses that working memory, processing speed, and digit span would be reduced in patients with CAH based on previous studies, these differences did not remain after we controlled for multiple comparisons (17, 32). Cognitive abnormalities in patients with CAH may relate to the supraphysiological glucocorticoid doses used to suppress adrenal androgen production (17). In support of this hypothesis working memory, episodic and declarative memory are adversely affected in patients treated with exogenous glucocorticoids, in adults with Cushing syndrome producing excess endogenous glucocorticoids, and in healthy human volunteers given glucocorticoids (33, 34). Our data provide further evidence that cognitive abnormalities present in patients with CAH relate to the degree of glucocorticoid exposure, with patients on higher current glucocorticoid doses having significantly worse performance on working memory and digit span tests.

We identified an increased prevalence of type 1 Chiari anomalies in patients with CAH, similar to a previous study of adults with CAH in which 8 of the 39 patients studied had type 1 Chiari anomalies (4). Chiari anomalies are a complex developmental disorder characterized by primary axial skeletal defects and secondary neurologic anomalies involving the craniocervical region (35). We found the cerebellum and hindbrain to be smaller in patients with CAH, suggesting that the downward displacement of the cerebellar tonsils does not relate to brain structural abnormalities. It is possible that in patients with CAH, exposure to steroid hormone abnormalities early in embryonic development may affect occipital bone formation antenatally (35). The relevance of this increased prevalence of type 1 Chiari anomalies to the clinical course and ongoing management in patients with CAH remains to be elucidated.

Patients with CAH are exposed to multiple potentially pathological hormone abnormalities, and the neural changes we describe are likely to be multifactorial. Hypoglycemia and salt loss at initial presentation and subsequently at the time of adrenal crisis are likely to affect brain structure. We were unable to examine the difference in CNS changes between patients with and without mineralocorticoid deficiency because of the small number of patients without salt loss recruited to the study. However, we identified no reduction in full-scale IQ in our patients with CAH, in contrast to previous studies that report reduced full-scale IQ in patients with a history of salt wasting crises necessitating inpatient hospital management (36). Future studies should aim to dissect the impact of salt wasting on the CNS by including patients with conditions such as isolated aldosterone synthase deficiency.

Gross structural brain white matter abnormalities have been described previously in patients with CAH, in conjunction with thinning of the corpus callosum and small volume hippocampi (4, 37). However, we have identified widespread abnormalities in white matter microstructure in patients with CAH. Because longitudinal DTI studies report corpus callosum FA to be higher in men than in women, our findings with regard to white matter microstructure are unlikely to be caused by excess androgen exposure (38). Animal and human disease models provide some evidence that the abnormalities we describe relate to glucocorticoid treatment in patients with CAH. In animal models prolonged exposure to raised glucocorticoid concentrations leads to the inhibition of oligodendrocyte precursor proliferation, reducing myelin production (39). Patients with Cushing disease have significant reductions in FA and increases in mean diffusivity throughout the brain (40). Importantly, partial resolution of neuritic degenerative changes in rodents, significant improvements in cognitive function, and reversal of cerebral atrophy in humans occur after withdrawal of glucocorticoids, suggesting that modification of treatment regimens in patients with CAH may improve myelination and cognitive function (41).

In contrast to the expected finding of increased amygdala volume in girls with CAH exposed to excess androgens antenatally, Merke *et al.* (28) identified smaller amygdala volumes by using a manual tracing method with volumetric MRI acquisition on a 1.5-T scanner. This finding was interpreted as an effect of postnatal excess glucocorticoid exposure in patients with CAH. Although we did not replicate this result, our findings of localized volume loss in the right hippocampus, the left and right thalami, the cerebellum, and the brainstem also differs from the pattern of larger limbic, insula, and occipital lobe volumes and the smaller parietal lobe and opercular left inferior frontal gyrus volumes seen in association with increased testosterone exposure (1, 13, 16, 36, 42).

Mineralocorticoid receptors, which have a similar affinity for cortisol and aldosterone, are located in limbic structures, particularly in the hippocampus, but are almost absent elsewhere in the brain (14). Neurons in the hippocampus do not express 11*β*-hydroxysteroid dehydrogenase type 2, which elsewhere in the body converts corticosterone and cortisol into inactive metabolites. Therefore, in the hippocampus, the mineralocorticoid receptor is considered mainly to be a glucocorticoid-activated receptor, making the hippocampus selectively vulnerable to nonphysiological fluctuations in glucocorticoid concentrations (14). Glucocorticoids increase excitatory amino acids and serotonin concentrations, leading to neural damage (33), and may also increase the vulnerability of neurons to other insults, such as ischemia, via reduced neuronal glucose uptake (43). Administration of oral hydrocortisone in humans leads to reduced hippocampal metabolism (44), and patients with Cushing syndrome, exposed chronically to high glucocorticoid concentrations, have localized volume loss in the hippocampus and temporal lobes (41). The cerebellum, which we found to be significantly smaller in patients with CAH, has the highest concentration of CNS glucocorticoid receptors (12), with exogenous glucocorticoids reducing cerebellum growth in mice (45). Although the current study design does not enable us to determine the exact etiology of the structural impairments identified, the reductions in neural volumes in regions of the brain with high concentrations of glucocorticoid receptors, in areas shown to be affected negatively by increased glucocorticoid exposure in *in vivo* and murine studies, suggests that the brain abnormalities we describe in patients with CAH relate to chronic excess glucocorticoid exposure. These findings require replication in larger, independent samples.

We identified a reduction in total choline, a marker of CNS myelination, inflammation, and membrane synthesis and repair in patients with CAH, which did not extend to the right parietal lobe. Exposure to higher glucocorticoid doses was significantly associated with mesial temporal lobe total choline. This finding suggests that reduction in total choline in the hippocampal voxel relates to raised glucocorticoid concentrations in patients with CAH because the mesial temporal lobe contains high concentrations of glucocorticoid receptors (2). Khiat *et al.* (15) identified a reduction in choline resonance in 13 patients with Cushing syndrome in frontal and thalamic areas, hypothesized to reflect an inhibition of phospholipase A2 activity by glucocorticoids; phospholipase A2 hydrolyzes specific ester bonds in membrane phospholipids (46). Importantly, after normalization of cortisol concentrations, improvements in memory and the brain metabolite profile were documented (15).

## Conclusion

CAH has a profound impact on normal brain and cognitive development, with the effects we describe most marked in the mesial temporal lobe. We also describe a significant association between current glucocorticoid replacement regimens and cognitive and CNS abnormalities. Although we are not able to control for previous episodes of hypoglycemia and salt loss at initial presentation and subsequently during adrenal crisis incidents, the recent development of more physiological glucocorticoid replacement regimens and other modalities that offer improved control of altered steroidogenesis in CAH (3) may provide opportunities to use the biomarkers identified in the current study (mean diffusivity, mesial temporal lobe total choline) to assess the impact treatments on the CNS in patients with CAH. These findings are also relevant to the wider population, of whom 1% are on long-term glucocorticoid therapy (47).

## Abbreviations:

21OHD: 21-hydroxylase deficiency
CAH: congenital adrenal hyperplasia
CNS: central nervous system
CSF: cerebrospinal fluid
DTI: diffusion tensor imaging
FA: fractional anisotropy
FDR: false discovery rate
Glx: glutamine plus glutamate
HADS: Hospital Anxiety and Depression Scale
MRI: magnetic resonance imaging
MRS: magnetic resonance spectroscopy
NAA: *N*-acetylaspartate and *N*-acetylaspartylglutamate
QOL: quality of life
SD: standard deviation
SE: standard error
WAIS: Wechsler Adult Intelligence Scale
WMS: Wechsler Memory Scale.
